# Rotational Diffusion of Soft Vesicles Filled by Chiral Active Particles

**DOI:** 10.1038/s41598-017-15095-0

**Published:** 2017-11-03

**Authors:** Jiamin Chen, Yunfeng Hua, Yangwei Jiang, Xiaolin Zhou, Linxi Zhang

**Affiliations:** Department of Physics, Zhejiang University, Hangzhou, 310027 China

## Abstract

We investigate the dynamics of two-dimensional soft vesicles filled with chiral active particles by employing the overdamped Langevin dynamics simulation. The unidirectional rotation is observed for soft vesicles, and the rotational angular velocity of vesicles depends mainly on the area fraction (ρ) and angular velocity (ω) of chiral active particles. There exists an optimal parameter for ω at which the rotational angular velocity of vesicle takes its maximal value. Meanwhile, at low concentration the continuity of curvature is destroyed seriously by chiral active particles, especially for large ω, and at high concentration the chiral active particles cover the vesicle almost uniformly. In addition, the center-of-mass mean square displacement for vesicles is accompanied by oscillations at short timescales, and the oscillation period of diffusion for vesicles is consistent with the rotation period of chiral active particles. The diffusion coefficient of vesicle decreases monotonously with increasing the angular velocity ω of chiral active particles. Our investigation can provide a few designs for nanofabricated devices that can be driven in a unidirectional rotation by chiral active particles or could be used as drug-delivery agent.

## Introduction

Active matter is a rapidly growing subject which has been studied theoretically and experimentally over the past few years^1–5^. A great many biological and physical systems can be referred to as active matter systems, including molecular motors^6^, swimming bacteria^7,8^, self-propelled colloids^9–11^, motile cells^12,13^, and macroscopic animals^14,15^. Previous works have demonstrated that active particles which perform spontaneous and sustained motion are fundamentally different from passive particles in thermal equilibrium systems. In other words, active matter systems move actively by gaining energy from an external source under non-equilibrium conditions. Hence, active particles with suitably designed constructions are able to convert energy into desired control of function, which have wide potential applications in a diversity of fields, such as drug delivery in medicine^16,17^. More details about microswimmers, propulsion mechanism and application prospects can be found in excellent review articles^18,19^.

In recent years, there are tremendous researches from experiments and computer simulations focused on the nanofabricated objects immersed in a bath of randomly swimming bacteria^20–22^, confirming that asymmetric environments can rectify the random motion of self-propelled particles and extract energy accordingly. That is, external confinement has a crucial impact on the motion of active particles. In addition, a great number of biological systems are confined in finite regions, leading to novel phenomena such as swarming^23–25^, swimming in a spiral vortex^26^, accumulating at walls^27,28^, and so on. Meanwhile, recent studies have also paid more attention to flexible boundaries enclosing active swimmers instead of rigid walls. For instance, the interesting case of active Brownian particles constrained within a deformable boundary has been deeply investigated, exhibiting obvious shape and displacement fluctuations in soft vesicles filled with active particles^29,30^.

As mentioned above, dynamics of self-propelled particles has captured the growing interest of various groups. In the models of self-propelled particles, it is assumed that the active particles possess uniaxial symmetry and that the direction of the self-propulsion is along the axis of symmetry of the particle. Thus active particles tend to orient along the active force and move in a straight line^31^. However, asymmetries in self-propulsion mechanism can generate a more complex behaviour, the direction of motion and that of the force are no longer aligned and the active particles tend to execute circular motion. Such chiral active particles have attracted mounting interest over the last few years^19,31–35^. Microscopy images show that non-tumbling *E. coli* bacteria swim in circular trajectories near planar glass surfaces^34^, indicating the motion of bacteria close to surfaces differs from the run-and-tumble motion in free solution. Active colloidal cell driven by gear-like spinners has also been observed^35^, which mainly focus on the shape control and compartmentalization of cells. Here, we choose spherical self-propelled and self-rotated particles without any special design as chiral active particles, and reveal more general organization principles of this class of active particles confined by a vesicle.

Active particles use a wide variety of mechanisms to achieve self-propulsion in liquid environments, and swimming at the macroscopic scale heavily relies on the inertia of the surrounding fluid. Hydrodynamic interactions between active particles and their detached vertical structures are the basis of higher swimming (or flying) efficiency^2,26^. In fact, short-range forces and noise dominate the interactions between swimming organisms, and the influence of hydrodynamic interactions is negligible^36^. Only for swimmers in close contact with a surface, the hydrodynamic effects become important, favouring long residences time along the walls. However, this effect is given also by self-propulsion^36^. Therefore, hydrodynamic interactions should not change the phenomenology qualitatively, and the active particles obey overdamped Langevin dynamics without the hydrodynamic interactions^2,29,37–42^. That is, we employ the overdamped Langevin dynamics simulations to study the dynamical behaviours of soft vesicles filled by spherical self-propelled and self-rotated particles. And the aim of this work is to explore how the additional angular velocity of chiral active Brownian particles affects the dynamics of soft vesicles. Owing to the presence of angular velocity for chiral active particles, active particles collide with the vesicle edge and consequently drive the vesicles into unidirectional rotation. The rotational angular velocity of vesicles depends mainly on the area fraction (ρ) as well as the angular velocity (ω) of chiral active particles. Meanwhile, the mean square displacement of the center of mass of vesicles filled with chiral active particles shows that the diffusion of vesicles is accompanied by the oscillation at short timescales, and its oscillation period is consistent with the rotation period of chiral active particles.

## Model and Methods

Molecular dynamics simulations are employed to study the dynamical behaviors of vesicles filled with chiral active particles. Chiral active particles are modelled as Lennard-Jones (LJ) spheres with a bead diameter of *σ*. Each chiral active particle moves with a translational velocity *V*_0_ for self-propulsion and a counterclockwise angular velocity *ω* for self-rotation, performing a circular active Brownian motion. The overdamped dynamics is governed by Langevin equations for the position *r*_*i*_ and the orientation *θ*_i_ of the polar axis ${\mathop{u}\limits^{\rightharpoonup }}_{i}$ = (cos*θ*_i_, sin*θ*_i_) of the center of the *i*-th active particle inside the vesicle^39–42^

1 $$d{\mathop{r}\limits^{\rightharpoonup }}_{i}/dt={V}_{0}{\mathop{u}\limits^{\rightharpoonup }}_{i}-(1/\gamma ){\nabla }_{i}U+\sqrt{{\rm{2}}{D}_{{\rm{0}}}}{\xi }_{i}^{T}(t)$$

2 $$d{\theta }_{i}/dt=\omega +\sqrt{2{D}_{\theta }}{{\xi }_{i}}^{R}(t)$$

where ${\mathop{r}\limits^{\rightharpoonup }}_{i}$ are the coordinates of particle, *γ* is the friction coefficient*, V*_0_ is the self-propulsion velocity of chiral active particles, and *U* is the configurational energy. *D*_*0*_ and *D*_*θ*_ denote the translational and rotational diffusion coefficients respectively. There exist the relations of γ = k_B_T/*D*_*0*_ and *D*_*θ*_ = 3*D*_*0*_/σ^2^ ^37–42^. ξ_i_^T^(t) and ξ_i_^R^(t) are Gaussian white noise with zero mean and satisfy < ξ_iα_^T^(t)ξ_jβ_^T^(s) > = δ_ij_δ_αβ_δ(t-s) (α,β = x,y) and < ξ_i_^R^(t)ξ_j_^R^(s) > = δ_ij_δ(t-s), respectively^43^. Here < …. > donates an ensemble average over the distribution of the noise, and δ is the Dirac delta function.

To prevent overlap, a shifted and cut-off Lennard-Jones potential *U*_*LJ*_ is used for chiral active particles as the configurational energy *U* in eqn (1)^44^,

3 $${U}_{LJ}(r)=\{\begin{array}{cc}4\varepsilon [{(\frac{\sigma }{r})}^{12}-{(\frac{\sigma }{r})}^{6}]+\frac{1}{4} & r < {2}^{1/6}\sigma \\ 0 & r\ge {2}^{1/6}\sigma \end{array}$$

Where *r* is the distance between any two active particles inside the vesicle, and ε = k_B_T.

The soft vesicle is modelled as a flexible ring polymer chain composed by *L* monomers whose diameter and mass are the same with the chiral active particles^29^. Here L represents the vesicle perimeter (i.e. the length of contour) in two-dimensional vesicle model. The equation of motion can also be described as eqn (1) with *V*_0_ = 0 for monomers of soft vesicles and the configurational potential energy consists of two parts:

4 $$U={U}_{LJ}+{U}_{bond}$$

The LJ potential is similar to eqn (3), and all neighboring monomers for vesicle interact with the bond potential^44^

5 $${U}_{bond}=\frac{{k}_{s}}{2}\sum _{i=1}^{L}{(|{r}_{i,i+1}|-{r}_{0})}^{2}$$

where *k*_*s*_ is the spring constant, and *r*_*i,i*+*1*_ is the distance between two neighbouring monomers. The parameters are chosen as *k*_*s*_ = 4000*k*_*B*_ *T/σ* ^2^, and *r*_0_ = σ.

The vesicle moves in the x-y plane of 200σ × 200σ with periodic boundary conditions. The initial configuration of the vesicle is a circle of radius R_0_ = (2sin(π/L))^−1^σ and the area fraction of chiral active particles for the initial vesicle is determined by ρ = Nσ^2^/(4R_0_^2^)^29^. Here N is the number of chiral active particles within the vesicle, and for example, N = 1596 for L = 150 and ρ = 0.7. MD simulations are carried out by performing Langevin dynamics with the open source software, LAMMPS^45^. We nondimensionalize the equations of motion using σ and ε as basic units of length and energy, and τ_0_ = σ^2^/*D*_0_ as the unit of time. And the time step is set to be τ = 0.0001 τ_0_. Meanwhile, the parameters are chosen to be *D*_*0*_ = 0.01, *D*_*θ*_ = 0.03, *γ* = 100, and *V*_*0*_ = 0.5. We have performed the simulations with various vesicle perimeters of L = 50, 100, and 150, and various area fractions of active particles from ρ = 0.05 to 0.7. Moreover, the angular velocities of chiral active particles are selected from ω = 0 to 1.0. The total simulation time for each run in our results is not less than 2 × 10^8^τ. In addition, all the simulation snapshots are captured using the Visual Molecular Dynamics (VMD) package^46^.

## Results and Discussion

### Rotation of the vesicle

**Figure 1:**
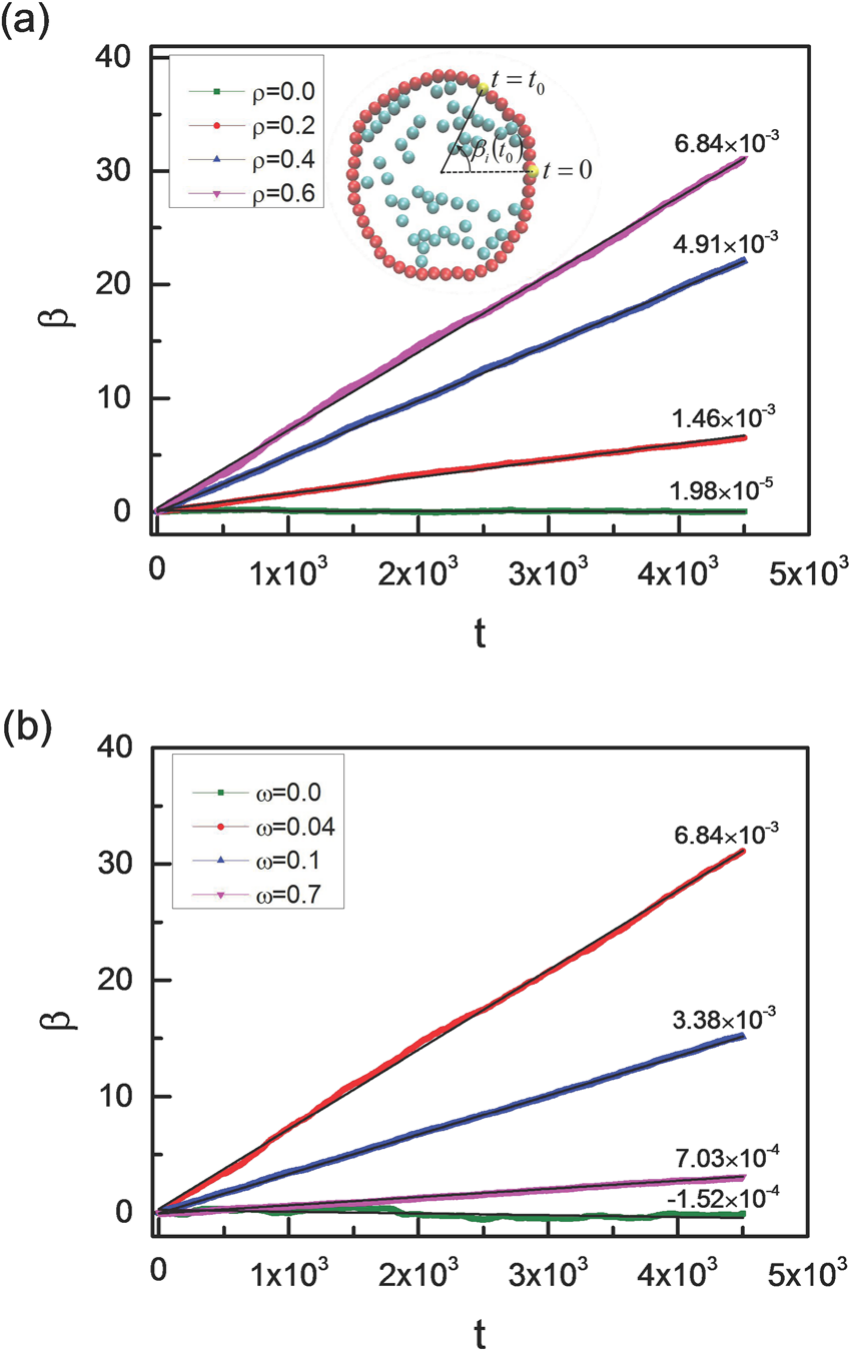
Rotation angle of the vesicle. Cumulative rotation angle β of a soft vesicle as a function of time *t* for various area fractions of active particles (ρ) with an angular velocity of ω = 0.04 (**a**) and for various angular velocities (ω) with an area fraction of ρ = 0.6 (**b**). The inset figure is a schematic illustration of the rotation angle β_i_(t_0_) of *i*-th monomer at time *t*_0_, and the straight black lines are linear fitting curves.

In our simulation, the chiral active particles perform the counterclockwise motion with ω > 0. These active particles can resemble molecular motors, and the collision with the vesicle membrane results in the rotational motion of vesicle in the same direction. Figure 1 shows that the rotation angle β of the vesicle increases gradually with time *t*. Here L = 50. The straight back lines are linear fitting curves and the slopes of these lines are also given in Fig. 1. The inset figure gives the definition of the rotation angle β_i_(t) for *i*-th monomer of the vesicle at time *t*. The average rotation angle β(t) for the vesicle is defined as

6 $$\beta (t)=\frac{1}{L}\sum _{i=1}^{L}{\beta }_{i}(t),$$

where β_i_(t) means the cumulative rotation angle of *i*-monomer at time *t*. Figure 1(a) presents the time evolution of the rotation angle β with various area fractions from ρ = 0 to 0.6 at a fixed angular velocity of chiral active particle of ω = 0.04. It shows a linearly dependence of the rotation angle β on time *t*, and the slope of line increases monotonically with ρ increasing. However, there is an exception for the case of ρ = 0, in which the cumulative rotation angle oscillates slightly near zero under thermal interference of a vesicle without any chiral active particles which can drive the vesicle to rotate counterclockwise. Moreover, we also study the dependence of cumulative angular angle β on time *t* with various angular velocities of chiral active particles, and the results are shown in Fig. 1(b). When the angular velocity of particles increases from ω = 0.0 to 0.7, the slope of line increases first and then decreases. Similarly, the rotation angle oscillates slightly near zero for ω = 0.0. Vesicle rotates more quickly for ω = 0.04 (see Supplementary Videos S1, S2, and S3).

**Figure 2:**
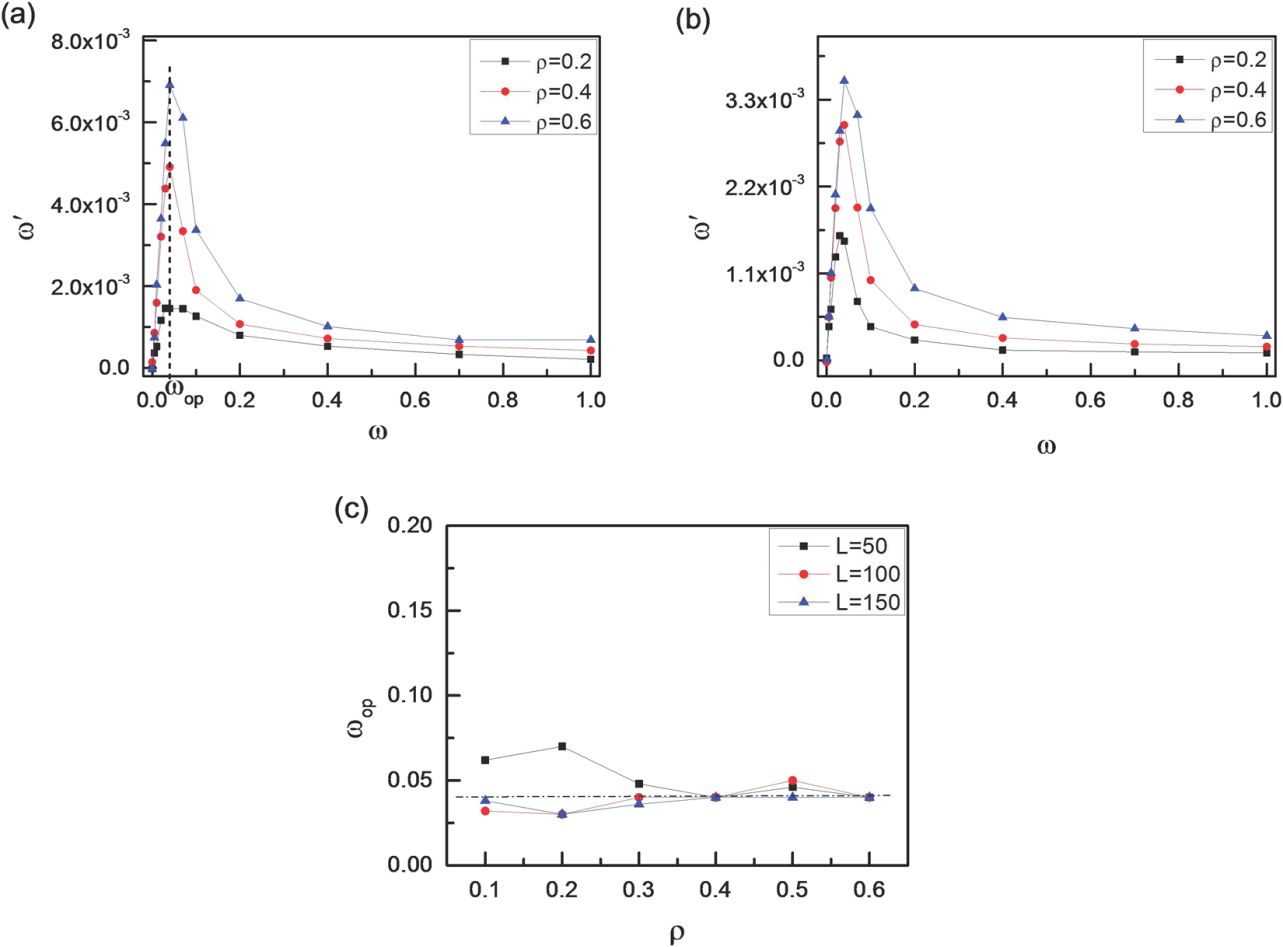
Rotation velocity of the vesicle. Average angular velocity of soft vesicle (ω’) as a function of rotational angular velocity of chiral active particles (ω) for three area fractions of ρ = 0.2, 0.4, and 0.6 with two vesicle perimeters of L = 50 (**a**) and L = 100(**b**). And (**c**) the optimum value of angular velocity (ω_op_) for chiral active particles as a function of area fraction ρ for three vesicle perimeters of L = 50, 100, and 150.

**Figure 3:**
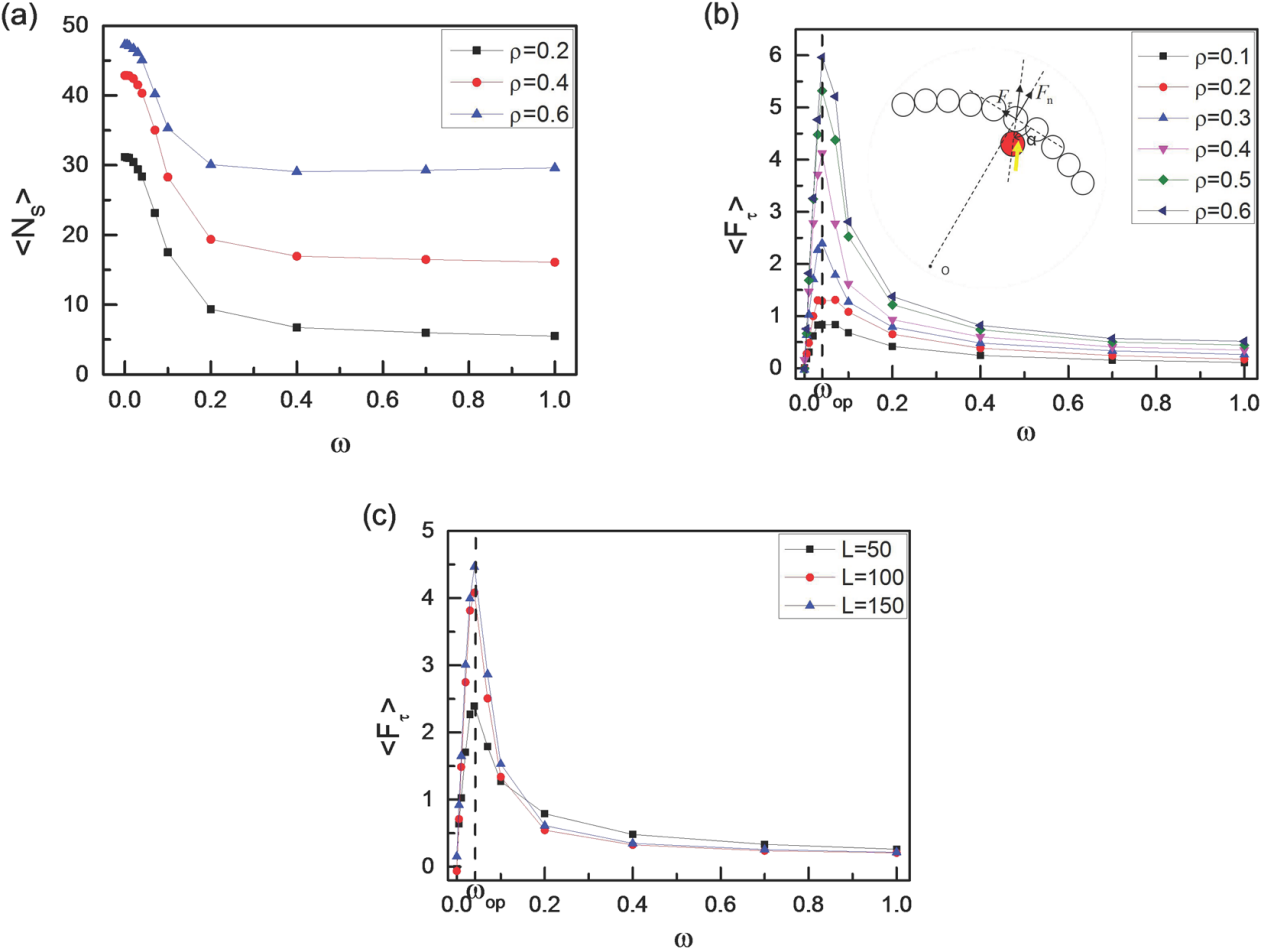
Average tangential force on membrane. (**a**) Average number of chiral active particles < N_S_ > stacked to the vesicle, and average tangential force < F_τ_ > per monomer of the vesicle exerted by chiral active particles as a function of angular velocity (ω) for various area fractions (ρ) with L = 50 (**b**) and for various vesicle perimeters (L) with ρ = 0.3 (**c**). The inset figure shows that the tangential (F_τ_) and normal forces (F_n_) rely on the collision angle α between the chiral active particles and the monomers of the vesicle.

**Figure 4:**
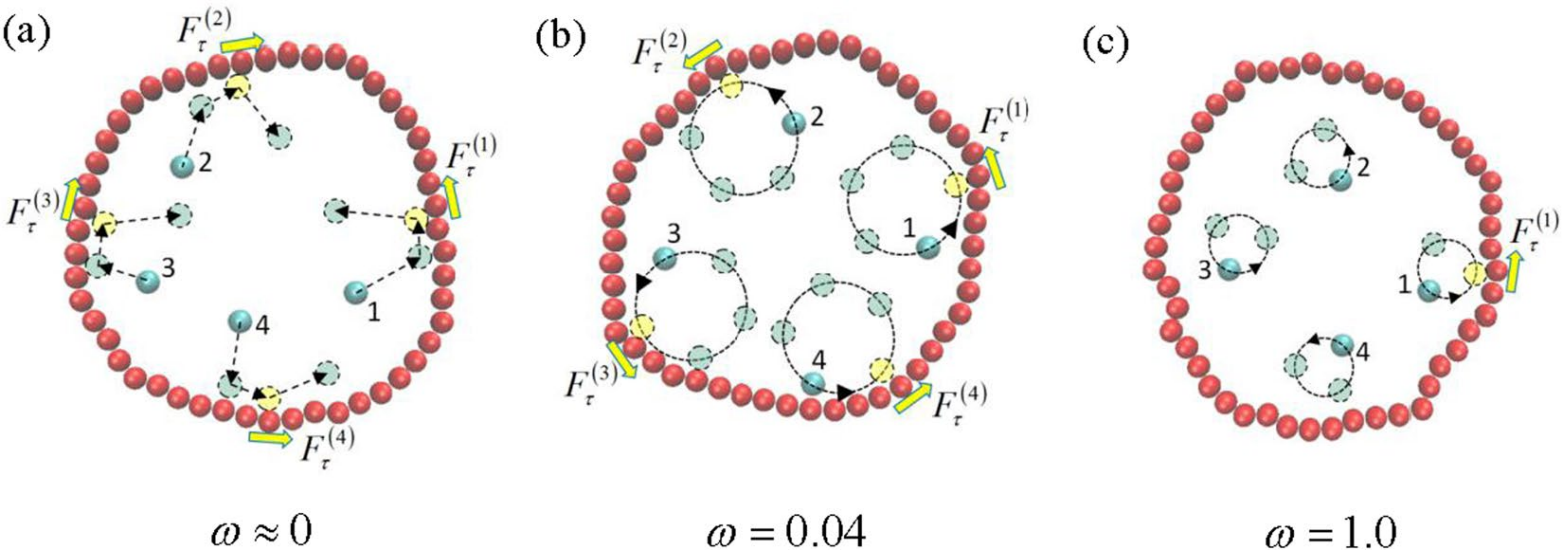
Schematic illustrations for the origin of unidirectional rotation. The rotation of soft vesicle is driven by the collisions between the chiral active particles and the membrane. Typical three cases of ω ≈ 0 (**a**), 0.04 (**b**) and 1.0(**c**) are considered, and Fig. 4(b) displays that the total counterclockwise force is large enough to drive the vesicle to rotate counterclockwise quickly because the tangential forces (F_τ_) produced by the collisions are more likely to be counterclockwise. While the counterclockwise tangential forces are often offset by the clockwise ones in Fig. 4(a) and the collisions with the membrane for chiral active particles occur rarely in Fig. 4(c).

In order to investigate the effects of the angular velocity ω on the rotational behavior of vesicles in more detail, we calculate the average angular velocity ω’ of vesicles according to

7 $$\omega ^{\prime} =\langle {\mathrm{lim}}_{t\to \infty }\frac{\beta (t)-\beta (0)}{t}\rangle $$

where < … > represents an ensemble average over more than 500 runs. The results with two different vesicle perimeters of L = 50 and 100 are given in Fig. 2(a) and (b). We observe sharp increase in the rotation angular velocity ω’ above ω = 0.04. Importantly, this angular velocity for chiral active particles with ω = 0.04 is almost the same for various area fractions of ρ = 0.2, 0.4 and 0.6, as well as for different vesicle perimeters of L = 50, and 100. Here, we define the optimum value of ω_op_ at which ω’ reaches its maximum value. Figure 2(c) shows that the optimum value of ω_op_ always appears about ω_op_ ≈ 0.04, which seems to be independent of the area fraction ρ and the vesicle perimeter L. In order to explain the novel non-monotonic behaviour of ω’ in Fig. 2(a) and (b), we calculate the average number < N_S_ > of chiral active particles stacked to the vesicle membrane, and the results are shown in Fig. 3(a). Obviously, few chiral active particles can be stacked to the membrane for larger angular velocity (ω). Actually, trajectories for chiral active particles are circular, and the radius of circular trajectory depends on mainly angular velocity of active particles ω^40^. The larger the angular velocity ω is, the smaller the radius of circular trajectory is^40^. Since the occurrence of collision with the membrane is rare for chiral active particles with large ω, the rotation of the vesicle is very slow for large ω, especially at a lower area fraction of ρ = 0.2. We also measure the average tangential force < F_τ_ > per monomer of the membrane with different angular velocities for various area fractions and various vesicle perimeters respectively, and the results are given in Fig. 3(b) and (c). The inset figure shows that the tangential force originates from the fact that the collision angle α between the active particles and the membrane isn’t always equal to π/2. In fact, α is inclined to be less than π/2 because of the counterclockwise rotation for chiral active particles. The curves of < F_τ_ > in Fig. 3(b) are in good agreement with that of ω’ in Fig. 2(a), and they have the same optimum angular velocity (ω_op_ ≈ 0.04). Therefore, the average tangential force < F_τ_ > plays an important role in the appearance of optimum angular velocity. By means of the schematic illustrations in Fig. 4, we can know clearly that the rotation of soft vesicle relies mainly on the angular velocity of chiral active particles. Three cases of ω ≈ 0, 0.04, and 1.0 are considered in Fig. 4. At a very low angular velocity of ω ≈ 0, the motion of active particles is dominated by the translational motion, leading to the disorganized collisions between the active particles and the membrane, see Fig. 4(a). Although the probability of collisions with the membrane is large enough for active particles, the rotation angular velocity ω’ of the vesicle is close to zero because some tangential forces are counterclockwise (i.e., ${F}_{\tau }^{1}$ and ${F}_{\tau }^{4}$) while the others are clockwise (i.e., ${F}_{\tau }^{2}$ and ${F}_{\tau }^{3}$). And the counterclockwise tangential forces are often offset by the clockwise ones, leading to a very small value of total tangential forces for the vesicle. In fact, if the active particles only consist of propelling force, the irreversible chaotic motion of active particles can be rectified only by asymmetric boundary^47^. For self-propelled active particles, the asymmetry boundary is a basic ingredient, as observed in many other thermal ratchet mechanisms^48^, and self-starting micromotors with asymmetric boundary can be observed in a bacterial bath^47^. However, at a moderate angular velocity of ω = 0.04, there exists the higher collision probability with the membrane for chiral active particles because the radius of circular trajectory for chiral active particles is very large and the chiral active particles usually are aggregated near the membrane, as shown in Fig. 3(a). Importantly, the collisions between the chiral active particles and the membrane lead to counterclockwise tangential forces (see Fig. 4(b)), and the total counterclockwise force is large enough to drive the vesicle to rotate counterclockwise quickly. Synchronization plays a role in the appearance of optimum angular velocity because the maximum value for average tangential force < F_τ_ > or rotation angular velocity ω’ only exists for high collision synchronization and high collision probability. As for the case of ω = 1.0, since the radius of circular trajectory for chiral active particles is fairly small, the collisions with the membrane occur rarely. Consequently, although the collision also produces the counterclockwise tangential force (see Fig. 4(c)), the total counterclockwise tangential force for the vesicle is very small, resulting in the low angular velocity for vesicle. The real origin of the unidirectional rotation of the vesicle is the circular motion of active chiral particles. The counterclockwise circular motion biases the collision angle between the active particles and vesicle and hence induces the unidirectional rotation of the vesicle.

### Shape properties

**Figure 5:**
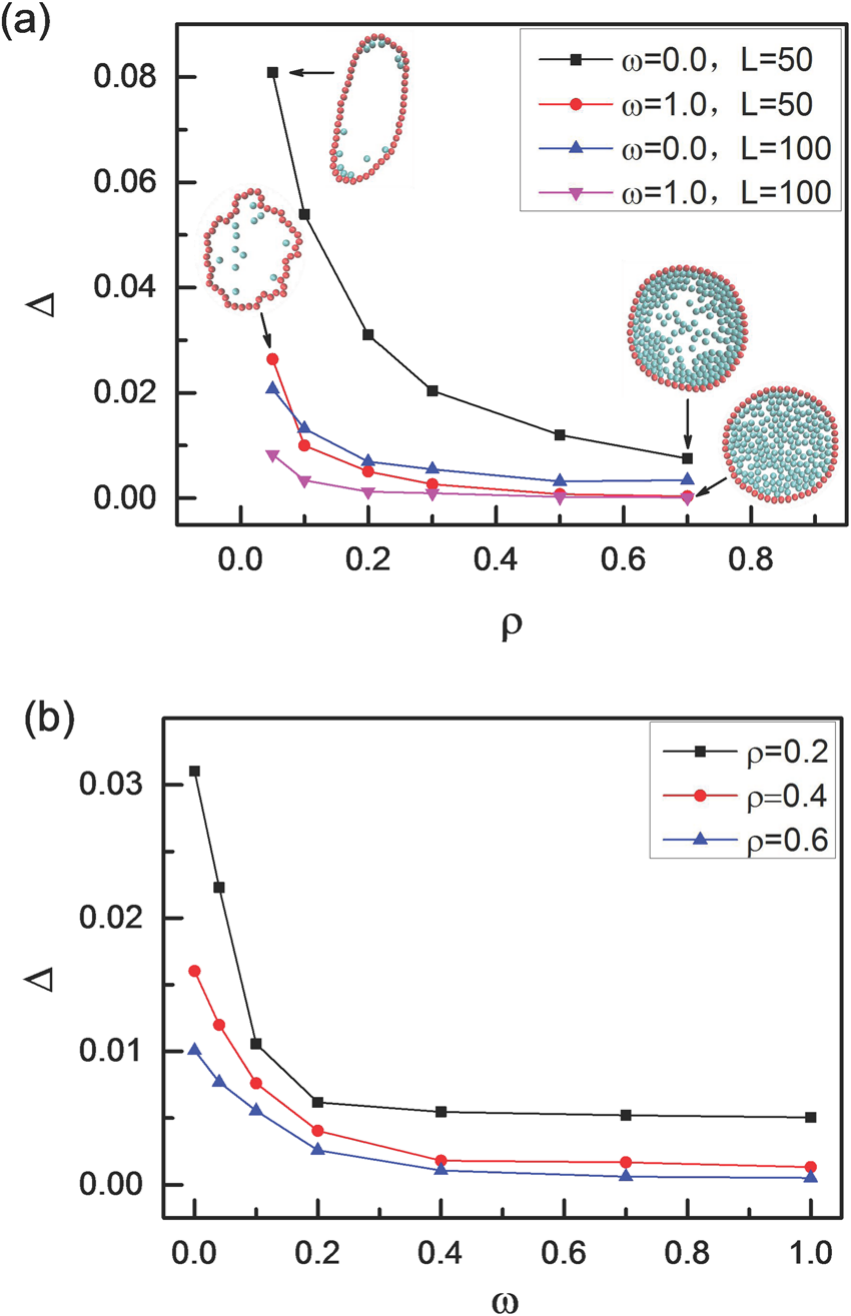
Asphericity of the vesicle. (**a**) Asphericity Δ of vesicle membrane as a function of area fraction ρ of active particles for two angular velocities ω and two vesicle perimeters L, and (**b**) asphericity Δ of vesicle membrane as a function of angular velocity ω of active particles for three area fractions of ρ = 0.2, 0.4, and 0.6 with L = 50. The inset figures show the configurations of the vesicles with different densities and different angular velocities.

**Figure 6:**
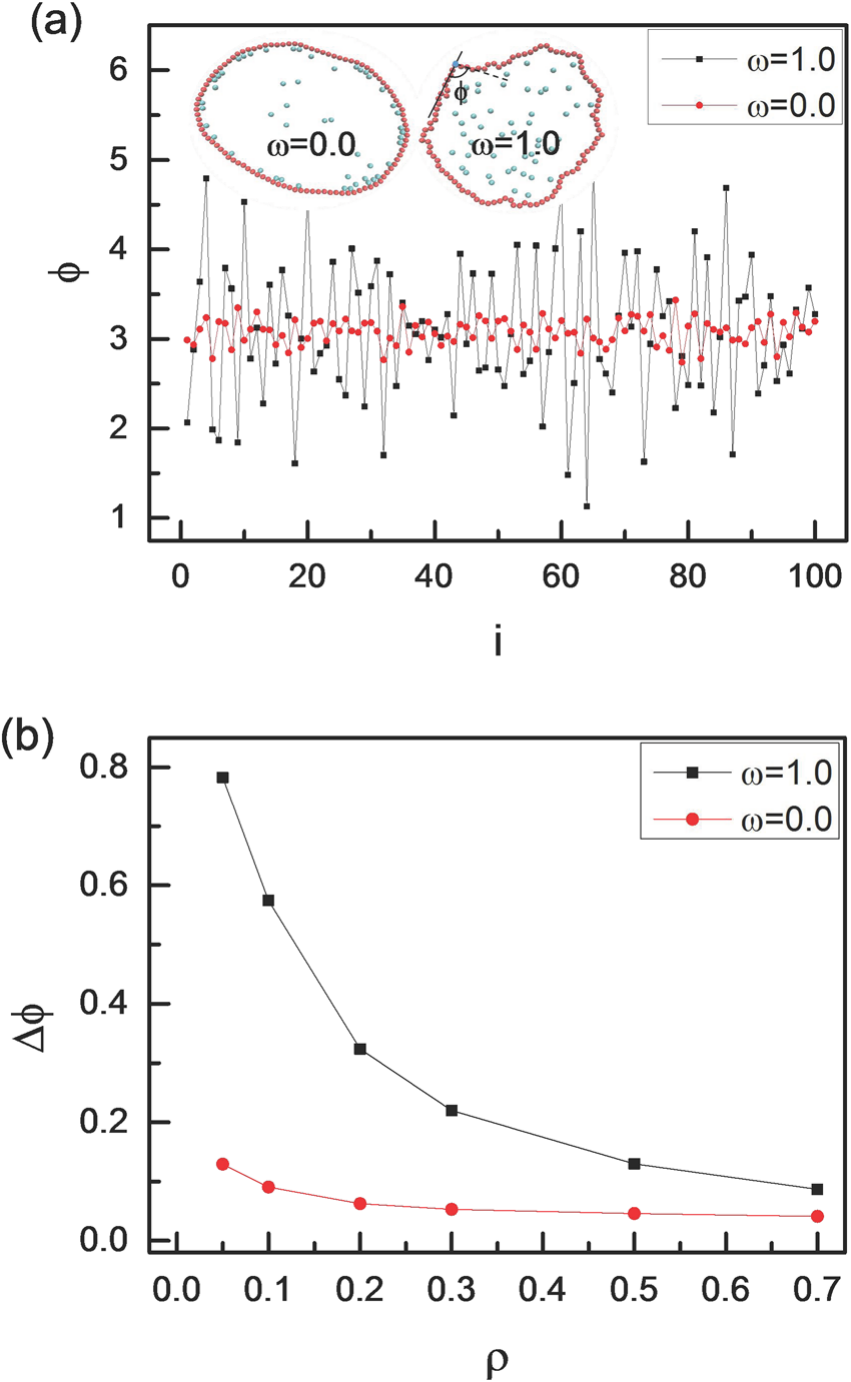
Fluctuation of interior angles for vesicle. (**a**) The interior angle ϕ of vesicle membrane for two angular velocities with ρ = 0.05, and (**b**) standard deviations ΔФ as a function of area fraction ρ for two angular velocities of ω = 0.0 and 1.0. Here L = 100.

**Figure 7:**
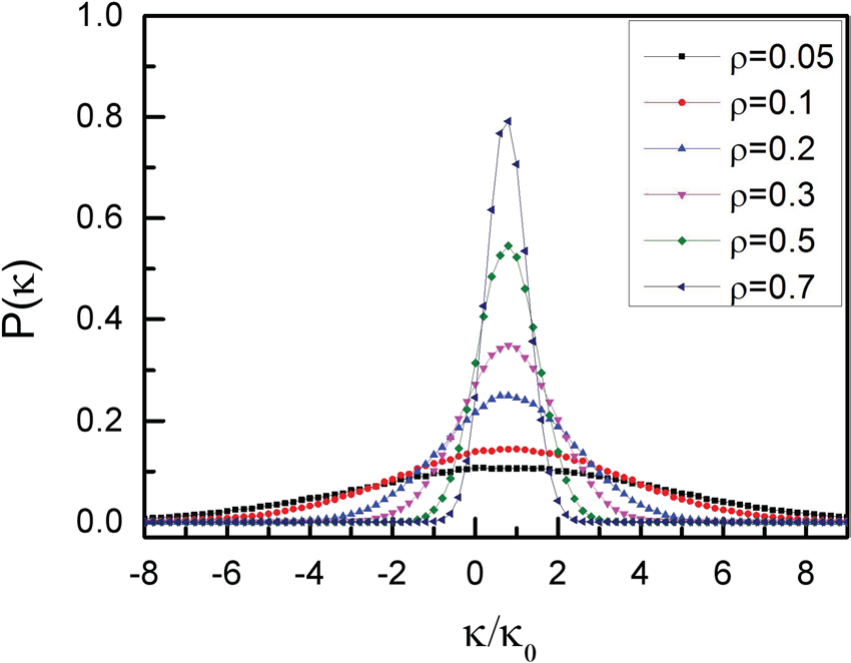
Local curvature of the vesicle. Probability distributions of the reduced local curvatures P(κ) for various area fractions of chiral active particles with an angular velocity of ω = 1.0 and L = 50.

To analyze the deformation of vesicle shape, we quantify the asphericity of vesicle (Δ)^49^, which is given by

8 $${\rm{\Delta }}=\frac{{({\lambda }_{{\rm{1}}}-{\lambda }_{{\rm{2}}})}^{{\rm{2}}}}{{({\lambda }_{{\rm{1}}}+{\lambda }_{{\rm{2}}})}^{{\rm{2}}}}$$

where λ_1_ and λ_2_ are two eigenvalues of the gyration tensor. The value Δ = 0 corresponds to a circle and Δ = 1 to a rod^29,49^. We display the asphericity Δ of vesicle as a function of area fraction ρ of active particles with different vesicle perimeters (L) and different angular velocities (ω) in Fig. 5(a). The asphericity Δ decreases monotonically with ρ increasing from 0.05 to 0.7, especially for ω = 0 and L = 50, which is in accordance with the work of Paoluzzi *et al*.^29^. Four detailed configurations of vesicle are also shown in Fig. 5(a). At a low area fraction of ρ = 0.05 with ω = 0, the active particles without angular velocity make the vesicle like an ellipse with a large asphericity of Δ = 0.08, however, the asphericity Δ decreases abruptly to Δ = 0.028 for chiral active particles with ω = 1.0. Of course, the difference of the asphericity Δ in ω = 0 and 1.0 almost disappears for a high area fraction of ρ = 0.7. We also calculate the asphericity Δ of vesicles with different angular velocities ω and the results are shown in Fig. 5(b). The asphericity Δ also presents a monotonic decreasing trend with ω increasing from 0 to 1.0. In order to know the deformation of vesicle membrane clearly at the low area fraction, we measure the interior angle φ and the results are shown in Fig. 6(a). Apparently, the fluctuation of interior angles around the average value is slight for ω = 0, however, it is violent for ω = 1.0. There exists a large deformation for vesicle filled with self-propelled active particles (ω = 0), and since all active particles are gathered near to the vesicle edge, the interior angle φ changes continuously, which is different from the case of ω = 1.0. In fact, the oscillations of the interior angle Ф can be quantified by the standard deviation ΔФ, which is defined as

9 $${\rm{\Delta }}{\varphi }= < \sqrt{\frac{1}{L}({\sum }_{i=1}^{L}{({{\varphi }}_{i}-\bar{{\varphi }})}^{2})} > $$

where $\bar{{\varphi }}$ is the average interior angle. Figure 6(b) shows clearly that the oscillations of the interior angle Ф are violent for larger angular velocity ω and lower area fraction ρ according to the large values of ΔФ. In fact, the small value of ΔФ means that the oscillation of the interior angle Ф is weak. For example, ΔФ = 0.78 for ω = 1.0 and ρ = 0.05, while it decreases to ΔФ = 0.04 for ω = 0 and ρ = 0.7. For chiral active particles with a large angular velocity, the radius of circular trajectory is small enough, the collisions with the membrane for chiral active particles occur rarely. Once the collisions occur, the chiral active particles can drive against the membrane seriously, and this leads to a sharp oscillation for Φ at ω = 1.0. Of course, the oscillation disappears gradually when the area fraction of active particles increases. The shape of membrane can also be characterized by the probability distribution of the local curvatures as well, as shown in Fig. 7. Note that the abscissa stands for reduced local curvature κ/κ_0_, and probability distributions P(κ) are normalized. Here, κ_0_ represents the curvature of initial circular configuration, and κ is the local curvature^29^. The reduced local curvatures follow Gaussian distributions centered near κ/κ_0_ ≈ 1 with decreasing widths, as the area fraction ρ increases gradually. The chiral active particles are distributed homogeneously for ω = 1.0, and they slowly stretch the membrane with increasing ρ. Combining with Fig. 5(a), we observe that the membrane becomes smoother as Δ tends to zero. And the concentration of curvatures is a generic consequence of the approaching to circle for vesicle, which means that local curvatures get closer to the same value of κ_0_.

**Figure 8:**
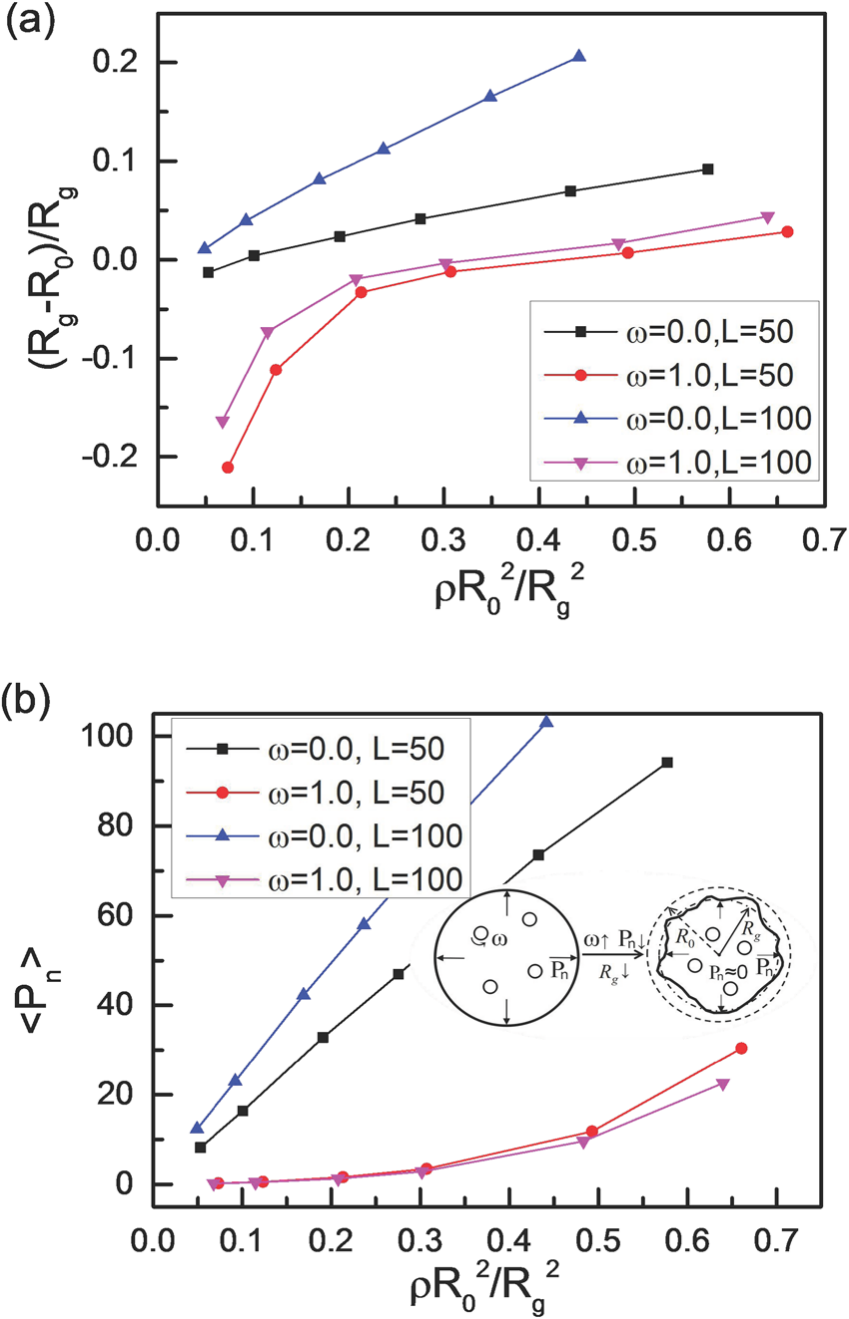
Average pressure on membrane. (**a**) The ratio of (R_g_–R_0_)/R_g_ and (**b**) average pressure < P_n_ > exerted by chiral active particles on the vesicle as a function of (ρR_0_^2^/R_g_^2^) for different angular velocities and different vesicle perimeters. The inset figure shows that the small pressure leads to the negative values of (R_g_–R_0_)/R_g_ for the vesicle.

Figure 8(a) shows the quantity (*R*_*g*_–*R*_0_)*/R*_*g*_ as a function of the actual area fraction ρR_0_^2^/R_g_^2^ with different vesicle perimeters. For active particles with ω = 0.0, (*R*_*g*_ *–R*_0_)*/R*_*g*_ scale linearly with the packing fraction, especially for L = 100. Deviations of the (*R*_*g*_ *–R*_0_)*/R*_*g*_ from the linear regime are visible at high area fraction ρ due to the excluded volume effects^50,51^. However, for vesicles filled with chiral active particles, most of the quantities (*R*_*g*_ *–R*_0_)*/R*_*g*_ are less than zero at the low packing fractions. In fact, (*R*_*g*_ *–R*_0_)*/R*_*g*_ is proportional to the average pressure exerted by the active particles^29,50,51^. We also measure the average pressure < P_n_ > exerted by the active particles on the membrane, and the results are shown in Fig. 8(b). For ω = 0, there exist large pressures exerted by the active particles, especially for higher area fractions. However, for ω = 1.0, the average pressure is very small, and it is close to zero for low area fractions owing to low contact probabilities between active particles and the membrane. And this leads to a decrease of R_g_ and produces a negative value of (R_g_–R_0_)/R_g_ for ω = 1.0, see the inset of Fig. 8(b). Therefore, the area fraction (ρ) and angular velocity (ω) of chiral active particles can affect the shape of vesicle seriously.

### Diffusion of the vesicle

Dynamical behaviors of vesicles are investigated through calculating the mean square displacement (MSD) of the center of mass of vesicles filled with chiral active particles, which is given by^52,53^.

10 $${g}_{3}(t)=\langle {({x}_{cm}(t)-{x}_{cm}(0))}^{2}+{({y}_{cm}(t)-{y}_{cm}(0))}^{2}\rangle $$

where x_cm_(t) and y_cm_(t) are the coordinates of the center of mass of vesicles filled with chiral active particles. Soft vesicles perform a random walk under the action of collision arising from chiral active particles. Since active particles and monomers of vesicle have the same size and mobility, the velocity of the center of mass ${\mathop{v}\limits^{\rightharpoonup }}_{cm}$ is given by^29^

11 $${\mathop{v}\limits^{\rightharpoonup }}_{cm}=\frac{1}{N+L}(\sum _{i=1}^{N}{\mathop{v}\limits^{\rightharpoonup }}_{i}+\sum _{n=1}^{L}{\mathop{v}\limits^{\rightharpoonup }}_{n})=\frac{\mu }{N+L}\sum _{i=1}^{N}{\mathop{f}\limits^{\rightharpoonup }}_{i}$$

where ${\mathop{v}\limits^{\rightharpoonup }}_{i}$ and ${\mathop{v}\limits^{\rightharpoonup }}_{n}$ are the velocities of an active particle and a membrane monomer, and *μ* is the mobility. The center of vesicle moves as a body of reduced mobility μ/(N + L) under the action of the total force on the active particles. The corresponding velocity-velocity correlation function is given by^29^

12 $$ < {\mathop{v}\limits^{\rightharpoonup }}_{cm}(t)\bullet {\mathop{v}\limits^{\rightharpoonup }}_{cm}(0) > =\frac{{\mu }^{2}}{{(N+L)}^{2}}\sum _{i,j}^{N} < {\mathop{f}\limits^{\rightharpoonup }}_{i}\bullet {\mathop{f}\limits^{\rightharpoonup }}_{j} > $$

For chiral active particles with an angular velocity of ω, forces are uncorrelated and the corresponding force-force correlation function is

13 $$\sum _{i,j}^{N} < {\mathop{f}\limits^{\rightharpoonup }}_{i}\bullet {\mathop{f}\limits^{\rightharpoonup }}_{j} > =N < \mathop{f}\limits^{\rightharpoonup }(0)\bullet f(t) > =\frac{N{V}_{0}^{2}}{{\mu }^{2}}{e}^{-{D}_{\theta }|t|}\,\cos (\omega |t|)$$

If ω = 0, eqn (13) becomes the Paoluzzi’s results^29^. Here, we also neglect the translational noise for the direct comparisons with the theoretical results in our simulation. The mean square displacement (MSD) of the center of mass of vesicles is calculated by a double time integration of eqn (13)^29^

14 $$\begin{array}{rcl}{g}_{3}(t) & = & \frac{N{V}_{0}^{2}}{{(N+L)}^{{\rm{2}}}}{\int }_{0}^{t}dt^{\prime} {\int }_{0}^{t}dt^{\prime\prime} {e}^{-{D}_{\theta }|t^{\prime} -t^{\prime\prime} |}\,\cos (\omega |t^{\prime} -t^{\prime\prime} |)\\  & = & \frac{{\rm{2}}N{V}_{0}^{2}}{({D}_{\theta }^{2}+{\omega }^{2})\,{(N+L)}^{2}}[{D}_{\theta }t+{e}^{-{D}_{\theta }t}\,\cos (\omega t+{\phi }_{0})-\,\cos \,{\phi }_{0}]\\  & = & 4Dt+A\lfloor {e}^{-{D}_{\theta }t}\,\cos (\omega t+{\phi }_{0})-\,\cos \,{\phi }_{0}\rfloor \end{array}$$

The parameter *A*, *D* and cos *φ*_0_ are given by

15 $$A=\frac{{\rm{2}}N{V}_{0}^{2}}{({D}_{\theta }^{2}+{\omega }^{2}){(N+L)}^{2}},$$

16 $$D=\frac{1}{4}A\times {D}_{\theta },$$

17 $$\cos \,{\phi }_{0}=\frac{{D}_{\theta }^{2}-{\omega }^{2}}{{D}_{\theta }^{2}+{\omega }^{2}}$$

Comparing with the theoretical results given by eqn (14), we find there exists some deviations from our simulation results at long timescale, especially for large ω. However, our simulation results show that the mean square displacement g_3_(t) follows the expression very well,

18 $${{\rm{g}}}_{3}(t)=4{D}_{eff}t+A\lfloor {e}^{-{D}_{\theta }t}\,\cos (\omega t+{\phi }_{0})-\,\cos \,{\phi }_{0}\rfloor $$

**Figure 9:**
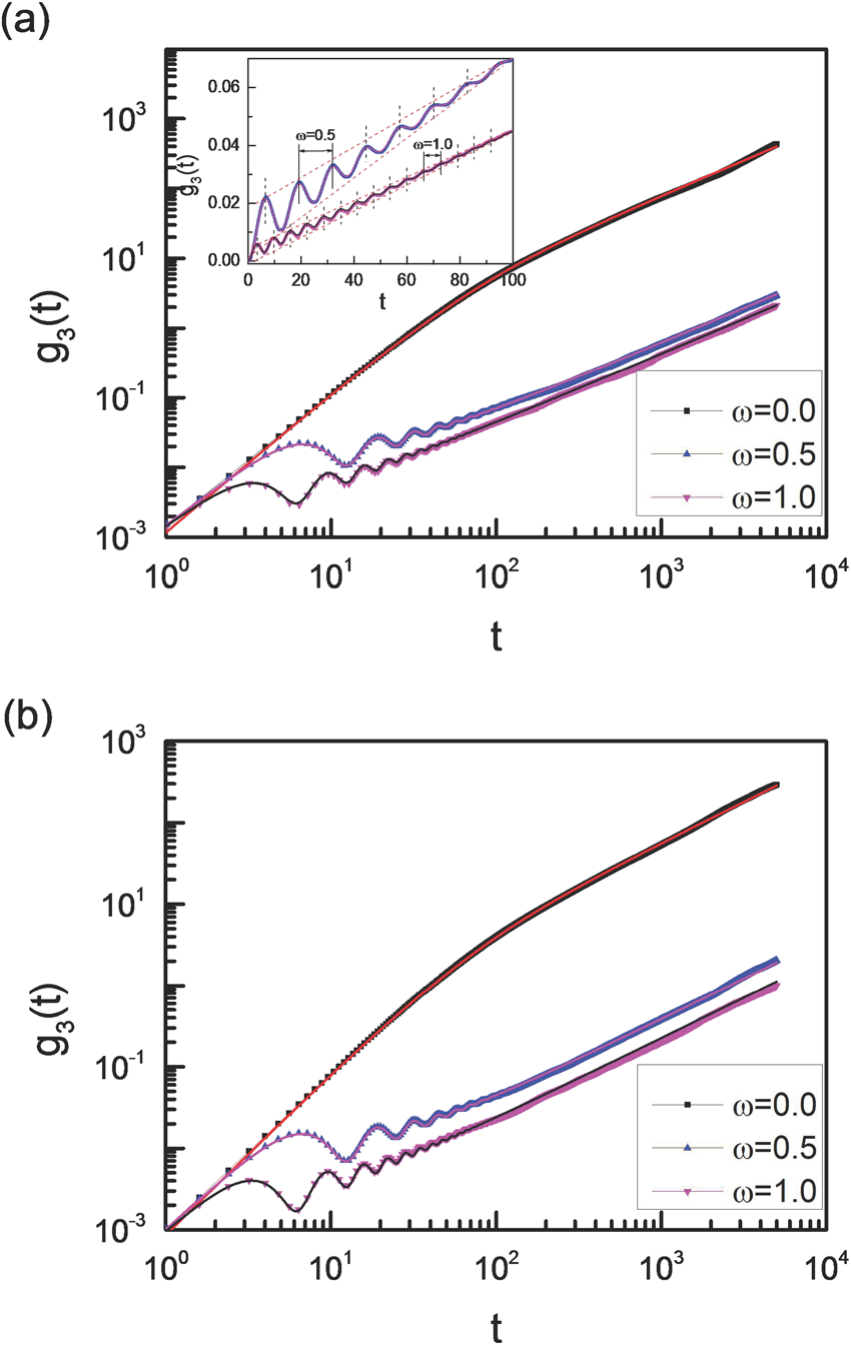
Diffusion of the vesicle. Mean square displacement g_3_(t) of the center of mass of vesicle for different angular velocities with two area fractions of ρ = 0.3 (**a**) and 0.7 (**b**). Here L = 50. Solid lines are fitting curves based on eqn (18) with the parameter of D_eff_ = 0.020 for ω = 0(red line), D_eff_ = 1.53 × 10^−4^ for ω = 0.5(pink line), and D_eff_ = 1.06 × 10^−4^ for ω = 1.0 (black line) in Fig. 9(a), and D_eff_ = 0.014 for ω = 0(red line), D_eff_ = 9.54 × 10^−5^ for ω = 0.5(pink line), and D_eff_ = 5.59 × 10^−5^ for ω = 1.0(black line) in Fig. 9(b). The inset figure shows that the oscillation period of the curve is consistent with the rotation period of chiral active particles, and the amplitude of oscillation decreases gradually with time *t*.

The values of *A* and cos*φ*_0_ are given by eqns (15) and (17), and D_eff_ is the fitting parameter. The fitting values of D_eff_ are also given in Fig. 9. In fact, D_eff_ is the effective diffusion coefficient over the classical value^9^. For ω = 0, a vesicle displays a superdiffusive motion at short timescales, while it recovers a normal diffusion at long timescale^54^. For ω ≠ 0, the diffusion of a vesicle is accompanied by oscillations at short timescales, and the oscillation period is consistent with the rotation period of chiral active particles, see the inset figure in Fig. 9. Meanwhile, the amplitude of oscillation decreases with time *t*, and the rotation motions of chiral active particles lead to the oscillation diffusion of vesicles. Moreover, the diffusion slows down significantly with the additional angular velocity for chiral active particles by about two orders, especially for a high area fraction of ρ = 0.7, see Fig. 9(b).

**Figure 10:**
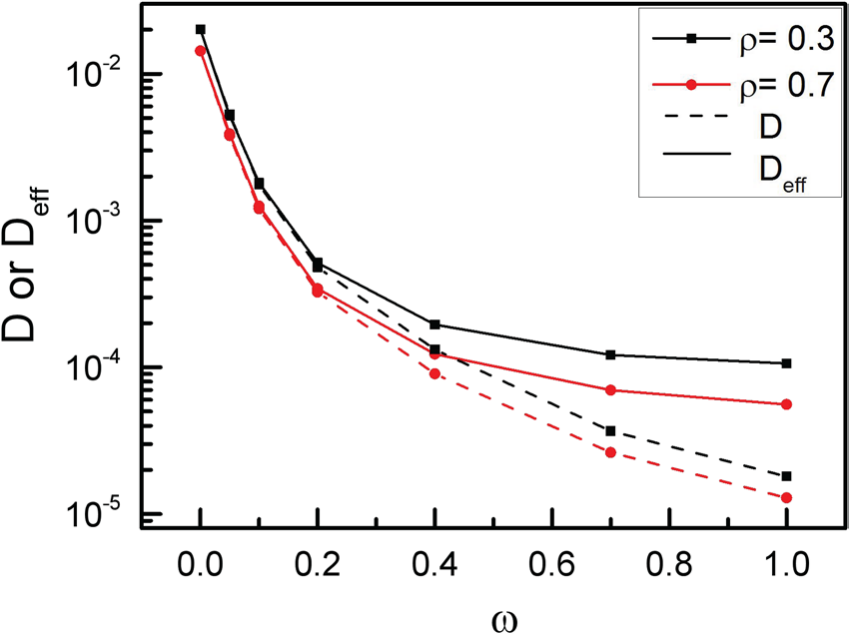
Diffusion coefficient. Both D and D_eff_ as a function of angular velocity ω for two area fractions of ρ = 0.3 and 0.7. Here D is given by eqn (16), D_eff_ is the fitting value, and L = 50.

A comparison between *D* and *D*_*eff*_ is made in Fig. 10. In general, the effects of angular velocity ω for chiral active particles on *D* and *D*_*eff*_ are obvious. However, *D*_*eff*_ is in good agreement with the theoretical results of *D* for ω < 0.2. In fact, for ω = 0, eqn (18) becomes a following expression

19 $${{\rm{g}}}_{3}(t)=4Dt+A({e}^{-{D}_{\theta }t}-1)$$

which is in agreement with the Paoluzzi’s results^29^. Both *D* and *D*_*eff*_ decrease abruptly with the angular velocity ω increasing for ω < 0.2, and slowly for ω > 0.7, especially for ρ = 0.7. Since trajectories for chiral active particles are circular, the radius of circular trajectory mainly depends on angular velocity ω of chiral active particles^40^. If the angular velocity ω is large enough, the radius of circular trajectory is very small, and the chiral active particles diffuse very slowly. If ω→∞, the chiral active particle only rotates around its center position, and *D* (or *D*_*eff*_) is close to 0. Of course, the corresponding force-force correlation function of eqn (13) may be only an approximate expression for large ω, which yields the smaller value for theoretical results of *D* than that of *D*_*eff*_. Therefore, the effects of angular velocity ω for chiral active particles on the diffusion behaviors are serious.

### Concluding Remarks

We have investigated numerically the dynamical behaviors of vesicles filled with chiral active particles using 2D Langevin dynamics simulations. In fact, the behavior of vesicles filled with chiral active particles bears some resemblance with the directed migration of Eukaryotic cells, as observed in would healing assays or in the presence of chemotactic cues^55–57^. For instance, random walk has been used to model the migration of endothelial cells during tumour-induced angiogenesis (growth of new blood vessels)^58,59^. When active particles touches the membrane, although all interactions between active particles and monomers of the membrane are center to center, the collision angle α between the active particles and the monomers of the membrane isn’t always equal to π/2 because of the counterclockwise rotation for chiral active particles. Accordingly, there exists net tangential forces for monomers of vesicle, and the rotating movement for vesicle occurs. Meanwhile, rotational angular velocity of vesicle depends mainly on the angular velocity (ω) and area fraction (ρ) of chiral active particles, and there exists an optimal parameter for ω at which the rotational angular velocity of vesicle takes its maximal value. Our results highlight that asymmetric environments can produce a spontaneous and directional rotation of vesicles filled with chiral active particles. Moreover, the shape of vesicle also relies on the area fraction (ρ) and angular velocity (ω) of chiral active particles as well as the vesicle perimeter (L). At low concentration the continuity of curvature is destroyed seriously for chiral active particles with large ω, and at high concentration the chiral active particles cover the vesicle almost uniformly, resulting in fairly homogeneous curvature and nearly circular vesicle shape. The center-of-mass mean square displacement for vesicle is accompanied by oscillations at short timescales, and the oscillation period of diffusion is consistent with the rotation period of chiral active particles. Meanwhile, the diffusion slows down significantly with the additional angular velocity for chiral active particles by about two orders, especially for high concentration. Our investigation can provide a few designs for nanofabricated devices with asymmetric environments that can be driven in a unidirectional rotation by chiral active particles.

## Electronic supplementary material

Supplementary Information: Rotational Diffusion of Soft Vesicles Filled by Chiral Active Particles

Video S1. Diffusion behavior of a soft vesicle filled by active particles with ω=0.

Video S2. Diffusion behavior of a soft vesicle filled by chiral active particles with ω=0.7.

Video S3. Diffusion behavior of a soft vesicle filled by chiral active particles with ω=0.04.
