# Supplementary material for: Rotational Diffusion of Soft Vesicles Filled by Chiral Active Particles
Source: Sci Rep. 2017 Nov 3;7:15006. doi: 10.1038/s41598-017-15095-0 (PMC5670181; doi:10.1038/s41598-017-15095-0)
Supplement: Supplementary file 1 — Supplementary Information: Rotational Diffusion of Soft Vesicles Filled by Chiral Active Particles [file 41598_2017_15095_MOESM1_ESM.doc]

**Supplementary Information**

**Rotational Diffusion of Soft Vesicles Filled by Chiral Active Particles**

**Jiamin Chen, Yunfeng Hua, Yangwei Jiang, Xiaolin Zhou, & Linxi Zhang**

**Department of Physics, Zhejiang University, Hangzhou, 310027, China**

^*^Corresponding author. E-mail: [lxzhang@zju.edu.cn](mailto:lxzhang@zju.edu.cn).

Phone: 86-571-88483790

**Video S1.** **Diffusion behavior of a soft vesicle filled by active particle****s with** ω=0**.**

This video shows the diffusion process of a soft vesicle filled with active particles. Here ω=0, ρ=0.6 and L=50.

**Video S2. Diffusion behavior of a soft vesicle filled by chiral active particles with** ω=0.7**.**

This video shows the diffusion process of a soft vesicle filled with chiral active particles. Here ω=0.7, ρ=0.6 and L=50.

**Video S3. Diffusion behavior of a soft vesicle filled by chiral active particles with** ω=0.04**.**

This video shows the diffusion process of a soft vesicle filled with chiral active particles. Here ω=0.04, ρ=0.6 and L=50.
